# 2*H*-[1,3]Thia­zolo[5,4,3-*ij*]quinolin-3-ium chloride monohydrate

**DOI:** 10.1107/S2414314620014650

**Published:** 2020-11-10

**Authors:** Madeleine A. Ehweiner, Ferdinand Belaj, Nadia C. Mösch-Zanetti

**Affiliations:** a University of Graz, Institute of Chemistry, Schubertstr. 1, 8010 Graz, Austria; University of Aberdeen, Scotland

**Keywords:** crystal structure, quinoline, hydrogen bonding

## Abstract

The structure of the title hydrated mol­ecular salt, C_10_H_8_NS^+^·Cl^−^·H_2_O, obtained by the reaction of sodium quinoline-8-thiolate Na(Quin-8-S) with CH_2_Cl_2_ and an aqueous solution of [Bu_4_N]Cl, contains π-stacked cations [plane-to-plane separation = 3.338 (4)–3.356 (4) Å] and features chains built by alternating Cl^−^ anions and H_2_O mol­ecules connected by O—H⋯O hydrogen bonds. The cation shows whole-mol­ecule disorder over two flipped orientations in a 0.853 (3):0.147 (3) ratio.

## Abstract 2

The cations in the title salt show π-stacking. The chloride anions, together with the water mol­ecules, form hydrogen-bonded zigzag chains.

## Graphical abstract

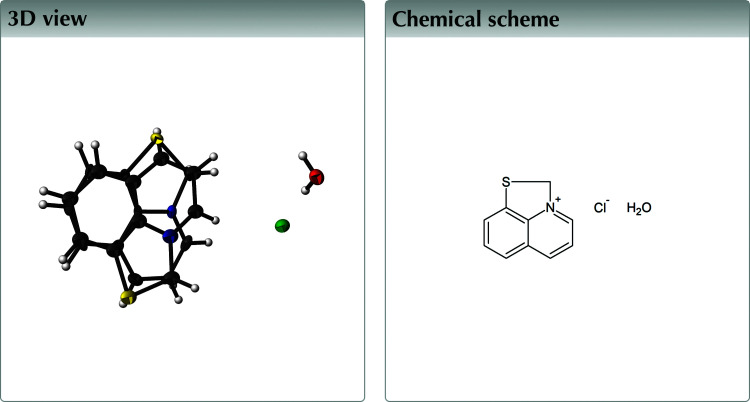

## Structure description

**Figure 1:**
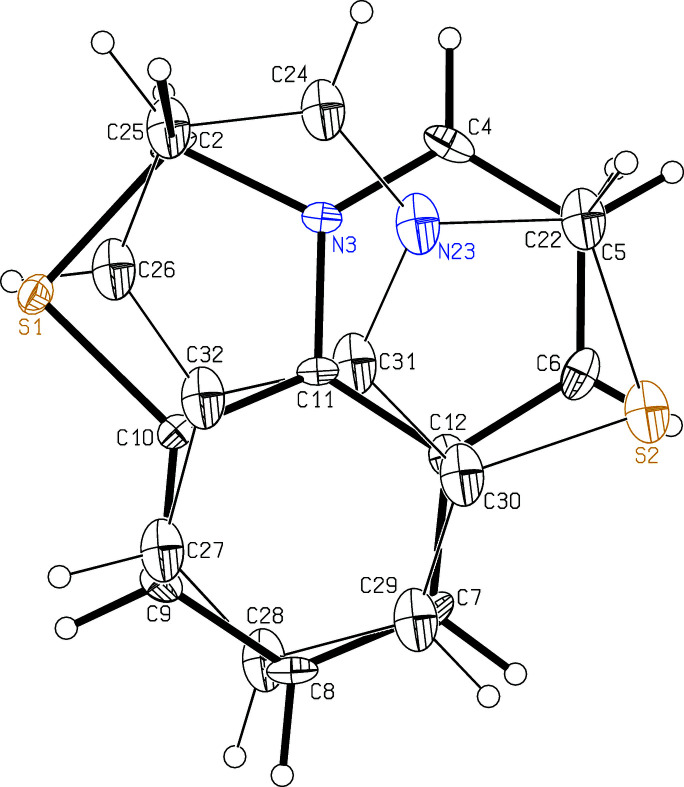
The mol­ecular structure of the disordered cation of the title compound. The bonds of the minor disorder component [14.7 (3)%] are drawn with thin lines. The probability ellipsoids are drawn at the 30% probability level, the H atoms are drawn with arbitrary radii.

**Figure 2:**
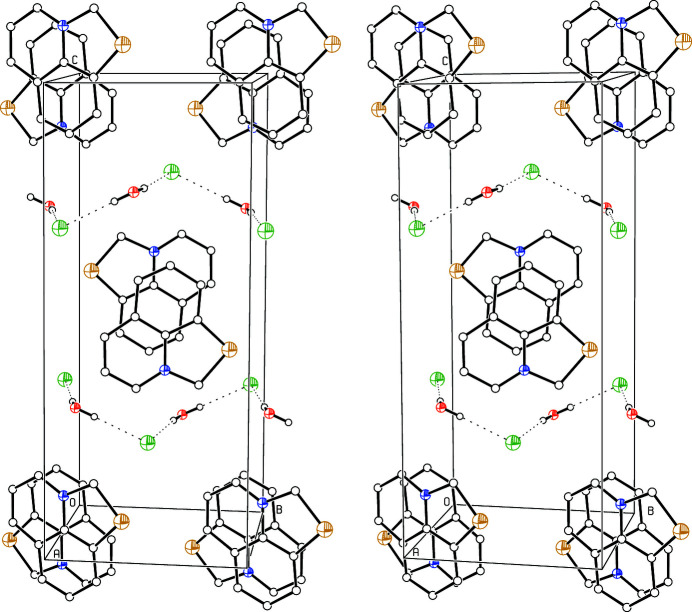
Stereoscopic *ORTEP* plot (Johnson, 1965 ▸) of the packing. The atoms are drawn with arbitrary radii. The cations in the less occupied orientations and the H atoms of the cations were omitted for clarity. The hydrogen bonds are indicated by dotted lines.

The crystal structure analysis of the title compound is the first structure determination of this tricyclic cation. The anhydrous iodide compound has previously been synthesized (Kim *et al.*, 1993 ▸). All atoms lie on general positions but the cation is planar within experimental accuracy. It is disordered over two orientations with occupation factors of 0.853 (3) and 0.147 (3) that both occupy approximately the same space (Fig. 1 ▸). The cations show π-stacking in the *a-*axis direction (Fig. 2 ▸), with the cations and inversion centers alternating. The distances between their least-squares planes are alternately 3.338 (4) and 3.356 (4) Å. The chloride anions, together with the water mol­ecules, form O—H⋯Cl hydrogen bonded (Table 1 ▸) zigzag chains running parallel to the *b* axis (Fig. 2 ▸).

## Synthesis and crystallization

During an attempt to obtain [Bu_4_N][Quin-8-S], an aqueous solution of [Bu_4_N]Cl was added to an aqueous solution of Na(Quin-8-S). The solution was then extracted with CH_2_Cl_2_ giving a yellow organic phase, which was then evaporated yielding a yellow oil. After a few hours, yellow–orange crystals of 2*H*-[1,3]thia­zolo[5,4,3-*ij*]quinolin-3-ium chloride monohydrate had formed in the oil.

## Refinement

Crystal data, data collection and structure refinement details are summarized in Table 2 ▸. The thia­zolo-quinolinium cation is disordered over two orientations, which refined to site occupation factors of 0.853 (3) and 0.147 (3), respectively. The same anisotropic displacement parameters were used for the ring atoms of the less occupied orientation and the equivalent bonds were restrained to have the same lengths.

## Supplementary Material

Crystal structure: contains datablock(s) I. DOI: 10.1107/S2414314620014650/hb4368sup1.cif

Structure factors: contains datablock(s) I. DOI: 10.1107/S2414314620014650/hb4368Isup2.hkl

Supporting information file. DOI: 10.1107/S2414314620014650/hb4368Isup3.mol

CCDC reference: 2042568

Additional supporting information:  crystallographic information; 3D view; checkCIF report

## Figures and Tables

**Table 1:** Hydrogen-bond geometry (Å, °)

*D*—H⋯*A*	*D*—H	H⋯*A*	*D*⋯*A*	*D*—H⋯*A*
O1—H11⋯Cl1	0.84	2.34 (1)	3.174 (2)	174 (2)
O1—H12⋯Cl1^i^	0.84	2.40 (1)	3.240 (2)	178 (2)

Symmetry code: (i) 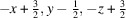 .

**Table 2:** Experimental details

Crystal data	
Chemical formula	C_10_H_8_NS^+^·Cl^−^·H_2_O
*M*_r_	227.70
Crystal system, space group	Monoclinic, *P*2_1_/*n*
Temperature (K)	100
*a*, *b*, *c* (Å)	7.0543 (11), 7.8252 (11), 18.223 (3)
β (°)	94.752 (7)
*V* (Å^3^)	1002.5 (3)
*Z*	4
Radiation type	Mo *K*α
μ (mm^−1^)	0.55
Crystal size (mm)	0.32 × 0.15 × 0.06

Data collection	
Diffractometer	Bruker APEXII CCD
Absorption correction	Multi-scan (*SADABS*; Bruker, 2001 ▸)
*T*_min_, *T*_max_	0.695, 1.000
No. of measured, independent and observed [*I* > 2σ(*I*)] reflections	6361, 1968, 1474
*R*_int_	0.056
(sin θ/λ)_max_ (Å^−1^)	0.617

Refinement	
*R*[*F* ^2^ > 2σ(*F* ^2^)], *wR*(*F* ^2^), *S*	0.042, 0.096, 1.04
No. of reflections	1968
No. of parameters	179
No. of restraints	16
H-atom treatment	Only H-atom displacement parameters refined
Δρ_max_, Δρ_min_ (e Å^−3^)	0.32, −0.30

Computer programs: *APEX2* and *SAINT* (Bruker, 2001 ▸), *SHELXS97* (Sheldrick, 2008 ▸), *SHELXL2014/6* (Sheldrick, 2015 ▸) and modified *ORTEP* (Johnson, 1965 ▸).

## full crystallographic data

### Crystal data

**Table:**

C_10_H_8_NS^+^·Cl^−^·H_2_O	*F*(000) = 472
*M**_r_* = 227.70	*D*_x_ = 1.509 Mg m^−^^3^
Monoclinic, *P*2_1_/*n*	Mo *K*α radiation, λ = 0.71073 Å
*a* = 7.0543 (11) Å	Cell parameters from 1699 reflections
*b* = 7.8252 (11) Å	θ = 2.8–26.1°
*c* = 18.223 (3) Å	µ = 0.55 mm^−^^1^
β = 94.752 (7)°	*T* = 100 K
*V* = 1002.5 (3) Å^3^	Plate, deep yellow
*Z* = 4	0.32 × 0.15 × 0.06 mm

### Data collection

**Table:**

Bruker APEXII CCD diffractometer	1968 independent reflections
Radiation source: Incoatec microfocus sealed tube	1474 reflections with *I* > 2σ(*I*)
Multilayer monochromator	*R*_int_ = 0.056
φ and ω scans	θ_max_ = 26.0°, θ_min_ = 2.2°
Absorption correction: multi-scan (SADABS; Bruker, 2001)	*h* = −8→8
*T*_min_ = 0.695, *T*_max_ = 1.000	*k* = −7→9
6361 measured reflections	*l* = −12→22

### Refinement

**Table:**

Refinement on *F*^2^	Primary atom site location: structure-invariant direct methods
Least-squares matrix: full	Secondary atom site location: difference Fourier map
*R*[*F*^2^ > 2σ(*F*^2^)] = 0.042	Hydrogen site location: mixed
*wR*(*F*^2^) = 0.096	Only H-atom displacement parameters refined
*S* = 1.04	*w* = 1/[σ^2^(*F*_o_^2^) + (0.026*P*)^2^ + 0.8115*P*] where *P* = (*F*_o_^2^ + 2*F*_c_^2^)/3
1968 reflections	(Δ/σ)_max_ = 0.001
179 parameters	Δρ_max_ = 0.32 e Å^−^^3^
16 restraints	Δρ_min_ = −0.30 e Å^−^^3^

### Special details

**Table:**

Geometry. All esds (except the esd in the dihedral angle between two l.s. planes) are estimated using the full covariance matrix. The cell esds are taken into account individually in the estimation of esds in distances, angles and torsion angles; correlations between esds in cell parameters are only used when they are defined by crystal symmetry. An approximate (isotropic) treatment of cell esds is used for estimating esds involving l.s. planes.
Refinement. Refinement of F^2^ against ALL reflections. The weighted R-factor wR and goodness of fit S are based on F^2^, conventional R-factors R are based on F, with F set to zero for negative F^2^. The threshold expression of F^2^ > 2σ(F^2^) is used only for calculating R-factors(gt) etc. and is not relevant to the choice of reflections for refinement. R-factors based on F^2^ are statistically about twice as large as those based on F, and R- factors based on ALL data will be even larger. The thiazolo-quinolinium cation was disordered over two orientations which refined to site occupation factors of 0.853 (3) and 0.147 (3), respectively. The same anisotropic displacement parameters were used for the ring atoms of the less occupied orientation (EADP of SHELXL) and the equivalent bonds were restrained to have the same lengths (SAME of SHELXL). The positions of the H atoms of the water molecule were taken from a difference Fourier map, the O-H distances were fixed to 0.84 Å, and the H atoms were refined with a common isotropic displacement parameter without any constraints to the bond angles. The H atoms of the CH_2_ groups were refined with a common isotropic displacement parameter and idealized geometries with approximately tetrahedral angles and C-H distances of 0.99 Å (AFIX 23 of SHELXL). The H atoms of the quinoline rings were put at the external bisectors of the C-C-C angles at C-H distances of 0.95 Å and a common isotropic displacement parameter was refined for these H atoms (AFIX 43 of SHELXL).

### Fractional atomic coordinates and isotropic or equivalent isotropic displacement parameters (Å^2^)

**Table:**

	*x*	*y*	*z*	*U*_iso_*/*U*_eq_	Occ. (<1)
S1	0.39598 (12)	0.13216 (12)	0.57556 (4)	0.0214 (3)	0.853 (3)
C2	0.3490 (11)	0.2798 (6)	0.6496 (2)	0.0205 (10)	0.853 (3)
H21	0.2482	0.2338	0.6788	0.029 (6)*	0.853 (3)
H22	0.4655	0.2976	0.6829	0.029 (6)*	0.853 (3)
N3	0.2867 (3)	0.4431 (4)	0.61387 (13)	0.0153 (6)	0.853 (3)
C4	0.2379 (4)	0.5808 (4)	0.65067 (19)	0.0233 (8)	0.853 (3)
H4	0.2434	0.5796	0.7029	0.027 (4)*	0.853 (3)
C5	0.1786 (10)	0.7272 (6)	0.61150 (19)	0.0234 (14)	0.853 (3)
H5	0.1436	0.8264	0.6372	0.027 (4)*	0.853 (3)
C6	0.1707 (6)	0.7285 (6)	0.5367 (2)	0.0246 (10)	0.853 (3)
H6	0.1309	0.8297	0.5111	0.027 (4)*	0.853 (3)
C7	0.2144 (18)	0.5684 (10)	0.4181 (3)	0.0204 (11)	0.853 (3)
H7	0.1756	0.6616	0.3870	0.027 (4)*	0.853 (3)
C8	0.2661 (13)	0.4161 (7)	0.3889 (3)	0.0208 (13)	0.853 (3)
H8	0.2660	0.4072	0.3369	0.027 (4)*	0.853 (3)
C9	0.3202 (9)	0.2702 (7)	0.43232 (19)	0.0200 (10)	0.853 (3)
H9	0.3497	0.1651	0.4098	0.027 (4)*	0.853 (3)
C10	0.3286 (7)	0.2850 (4)	0.50763 (17)	0.0149 (9)	0.853 (3)
C11	0.2783 (8)	0.4407 (5)	0.53779 (18)	0.0134 (10)	0.853 (3)
C12	0.2196 (16)	0.5844 (5)	0.4958 (3)	0.0183 (9)	0.853 (3)
Cl1	0.56970 (10)	0.56684 (9)	0.80204 (4)	0.0255 (2)	
O1	0.9855 (2)	0.4364 (2)	0.77707 (10)	0.0274 (5)	
H11	0.8735 (5)	0.4705 (12)	0.7800 (10)	0.037 (7)*	
H12	0.969 (3)	0.3406 (4)	0.7567 (4)	0.037 (7)*	
S2	0.1620 (9)	0.8059 (9)	0.5181 (4)	0.0328 (18)	0.147 (3)
C22	0.206 (9)	0.734 (3)	0.6134 (7)	0.0328 (18)	0.147 (3)
H221	0.3133	0.7991	0.6386	0.029 (6)*	0.147 (3)
H222	0.0920	0.7518	0.6404	0.029 (6)*	0.147 (3)
N23	0.254 (3)	0.550 (3)	0.6109 (10)	0.0328 (18)	0.147 (3)
C24	0.302 (3)	0.445 (2)	0.6669 (12)	0.0328 (18)	0.147 (3)
H24	0.2906	0.4853	0.7155	0.027 (4)*	0.147 (3)
C25	0.369 (8)	0.280 (4)	0.6575 (16)	0.0328 (18)	0.147 (3)
H25	0.4197	0.2139	0.6985	0.027 (4)*	0.147 (3)
C26	0.360 (3)	0.215 (3)	0.5881 (12)	0.0328 (18)	0.147 (3)
H26	0.3776	0.0953	0.5821	0.027 (4)*	0.147 (3)
C27	0.336 (7)	0.275 (6)	0.4491 (17)	0.0328 (18)	0.147 (3)
H27	0.3914	0.1696	0.4368	0.027 (4)*	0.147 (3)
C28	0.269 (9)	0.382 (6)	0.394 (3)	0.0328 (18)	0.147 (3)
H28	0.2510	0.3449	0.3440	0.027 (4)*	0.147 (3)
C29	0.228 (13)	0.553 (7)	0.414 (2)	0.0328 (18)	0.147 (3)
H29	0.2043	0.6360	0.3769	0.027 (4)*	0.147 (3)
C30	0.220 (12)	0.602 (3)	0.487 (2)	0.0328 (18)	0.147 (3)
C31	0.259 (7)	0.480 (5)	0.5414 (13)	0.0328 (18)	0.147 (3)
C32	0.327 (6)	0.317 (4)	0.5245 (14)	0.0328 (18)	0.147 (3)

### Atomic displacement parameters (Å^2^)

**Table:**

	*U*^11^	*U*^22^	*U*^33^	*U*^12^	*U*^13^	*U*^23^
S1	0.0242 (5)	0.0167 (5)	0.0228 (4)	0.0006 (4)	−0.0008 (3)	0.0036 (3)
C2	0.023 (3)	0.0264 (19)	0.0107 (16)	−0.0043 (16)	−0.0047 (16)	0.0083 (14)
N3	0.0105 (13)	0.0221 (15)	0.0134 (13)	−0.0035 (11)	0.0019 (10)	−0.0009 (11)
C4	0.0187 (18)	0.033 (2)	0.0190 (16)	−0.0096 (15)	0.0062 (14)	−0.0105 (15)
C5	0.014 (4)	0.0217 (19)	0.0346 (19)	−0.0044 (15)	0.0052 (15)	−0.0142 (15)
C6	0.0154 (19)	0.020 (3)	0.038 (2)	−0.0044 (18)	−0.0019 (16)	0.0085 (19)
C7	0.011 (3)	0.027 (3)	0.0225 (18)	−0.003 (2)	−0.0034 (15)	0.0107 (16)
C8	0.0154 (17)	0.037 (4)	0.0101 (15)	−0.005 (3)	0.0005 (13)	0.0014 (17)
C9	0.014 (2)	0.027 (2)	0.0187 (19)	−0.0008 (16)	−0.0056 (19)	−0.009 (2)
C10	0.0109 (15)	0.0163 (19)	0.0169 (19)	−0.0035 (16)	−0.0020 (17)	0.0015 (14)
C11	0.005 (2)	0.024 (3)	0.0111 (14)	−0.004 (2)	0.0006 (12)	0.0009 (13)
C12	0.0078 (15)	0.020 (2)	0.027 (2)	−0.005 (2)	−0.001 (2)	−0.0029 (17)
Cl1	0.0225 (4)	0.0236 (4)	0.0308 (4)	−0.0005 (3)	0.0038 (3)	−0.0017 (3)
O1	0.0209 (11)	0.0319 (12)	0.0291 (11)	0.0027 (9)	0.0003 (9)	0.0007 (9)
S2	0.015 (3)	0.028 (4)	0.054 (4)	0.001 (2)	−0.009 (2)	−0.006 (3)
C22	0.015 (3)	0.028 (4)	0.054 (4)	0.001 (2)	−0.009 (2)	−0.006 (3)
N23	0.015 (3)	0.028 (4)	0.054 (4)	0.001 (2)	−0.009 (2)	−0.006 (3)
C24	0.015 (3)	0.028 (4)	0.054 (4)	0.001 (2)	−0.009 (2)	−0.006 (3)
C25	0.015 (3)	0.028 (4)	0.054 (4)	0.001 (2)	−0.009 (2)	−0.006 (3)
C26	0.015 (3)	0.028 (4)	0.054 (4)	0.001 (2)	−0.009 (2)	−0.006 (3)
C27	0.015 (3)	0.028 (4)	0.054 (4)	0.001 (2)	−0.009 (2)	−0.006 (3)
C28	0.015 (3)	0.028 (4)	0.054 (4)	0.001 (2)	−0.009 (2)	−0.006 (3)
C29	0.015 (3)	0.028 (4)	0.054 (4)	0.001 (2)	−0.009 (2)	−0.006 (3)
C30	0.015 (3)	0.028 (4)	0.054 (4)	0.001 (2)	−0.009 (2)	−0.006 (3)
C31	0.015 (3)	0.028 (4)	0.054 (4)	0.001 (2)	−0.009 (2)	−0.006 (3)
C32	0.015 (3)	0.028 (4)	0.054 (4)	0.001 (2)	−0.009 (2)	−0.006 (3)

### Geometric parameters (Å, º)

**Table:**

S1—C2	1.827 (4)	O1—H12	0.84
S1—C10	1.758 (4)	S2—C30	1.758 (6)
N3—C2	1.484 (5)	S2—C22	1.827 (6)
C2—H21	0.99	C22—N23	1.484 (6)
C2—H22	0.99	C22—H221	0.99
N3—C4	1.329 (4)	C22—H222	0.99
N3—C11	1.383 (4)	N23—C24	1.329 (5)
C4—C5	1.395 (5)	N23—C31	1.384 (5)
C4—H4	0.95	C24—C25	1.395 (7)
C5—C6	1.360 (5)	C24—H24	0.95
C5—H5	0.95	C25—C26	1.360 (7)
C6—C12	1.409 (5)	C25—H25	0.95
C6—H6	0.95	C26—C32	1.411 (7)
C7—C8	1.366 (5)	C26—H26	0.95
C7—C12	1.420 (5)	C27—C28	1.366 (6)
C7—H7	0.95	C27—C32	1.419 (6)
C8—C9	1.423 (5)	C27—H27	0.95
C8—H8	0.95	C28—C29	1.423 (7)
C9—C10	1.374 (4)	C28—H28	0.95
C9—H9	0.95	C29—C30	1.373 (6)
C10—C11	1.394 (4)	C29—H29	0.95
C11—C12	1.403 (5)	C30—C31	1.394 (6)
O1—H11	0.84	C31—C32	1.402 (6)
			
N3—C2—S1	106.6 (2)	C30—S2—C22	90.3 (14)
N3—C2—H21	110.4	N23—C22—S2	106.9 (11)
S1—C2—H21	110.4	N23—C22—H221	110.3
N3—C2—H22	110.4	S2—C22—H221	110.3
S1—C2—H22	110.4	N23—C22—H222	110.3
H21—C2—H22	108.6	S2—C22—H222	110.3
C2—S1—C10	91.98 (16)	H221—C22—H222	108.6
S1—C10—C9	129.2 (3)	C24—N23—C31	116 (3)
S1—C10—C11	112.3 (2)	C24—N23—C22	128.2 (18)
C9—C10—C11	118.4 (4)	C31—N23—C22	116 (2)
C10—C11—N3	114.6 (3)	N23—C24—C25	123 (3)
C12—C11—N3	121.4 (3)	N23—C24—H24	118.5
C10—C11—C12	124.0 (3)	C25—C24—H24	118.5
C11—N3—C2	114.5 (3)	C26—C25—C24	118 (3)
C11—N3—C4	121.7 (3)	C26—C25—H25	121.0
C2—N3—C4	123.8 (3)	C24—C25—H25	121.0
N3—C4—C5	119.1 (4)	C25—C26—C32	123 (3)
N3—C4—H4	120.5	C25—C26—H26	118.5
C5—C4—H4	120.5	C32—C26—H26	118.5
C6—C5—C4	120.3 (4)	C28—C27—C32	122 (4)
C6—C5—H5	119.8	C28—C27—H27	118.8
C4—C5—H5	119.8	C32—C27—H27	118.8
C5—C6—C12	122.1 (4)	C27—C28—C29	116 (5)
C5—C6—H6	118.9	C27—C28—H28	121.9
C12—C6—H6	118.9	C29—C28—H28	121.9
C8—C7—C12	118.6 (6)	C30—C29—C28	123 (5)
C8—C7—H7	120.7	C30—C29—H29	118.6
C12—C7—H7	120.7	C28—C29—H29	118.6
C7—C8—C9	123.5 (6)	C29—C30—C31	118 (3)
C7—C8—H8	118.3	C29—C30—S2	127 (3)
C9—C8—H8	118.3	C31—C30—S2	115 (2)
C10—C9—C8	118.4 (5)	N23—C31—C30	112 (3)
C10—C9—H9	120.8	N23—C31—C32	127 (3)
C8—C9—H9	120.8	C30—C31—C32	121.1 (17)
C11—C12—C6	115.3 (4)	C31—C32—C26	112 (3)
C11—C12—C7	117.1 (4)	C31—C32—C27	118 (2)
C6—C12—C7	127.6 (5)	C26—C32—C27	130 (3)
H11—O1—H12	102.2 (17)		
			
C10—S1—C2—N3	−1.3 (5)	C30—S2—C22—N23	−3 (4)
S1—C2—N3—C4	179.3 (3)	S2—C22—N23—C24	178 (2)
S1—C2—N3—C11	0.8 (6)	S2—C22—N23—C31	2 (5)
C11—N3—C4—C5	−0.6 (6)	C31—N23—C24—C25	5 (5)
C2—N3—C4—C5	−179.0 (6)	C22—N23—C24—C25	−171 (5)
N3—C4—C5—C6	0.1 (8)	N23—C24—C25—C26	−9 (6)
C4—C5—C6—C12	0.5 (10)	C24—C25—C26—C32	14 (6)
C12—C7—C8—C9	1.9 (17)	C32—C27—C28—C29	14 (9)
C7—C8—C9—C10	−2.8 (13)	C27—C28—C29—C30	−12 (12)
C8—C9—C10—C11	1.9 (9)	C28—C29—C30—C31	1 (13)
C8—C9—C10—S1	−178.0 (5)	C28—C29—C30—S2	−177 (6)
C2—S1—C10—C9	−178.5 (6)	C22—S2—C30—C29	−177 (8)
C2—S1—C10—C11	1.6 (5)	C22—S2—C30—C31	4 (6)
C4—N3—C11—C10	−178.1 (4)	C24—N23—C31—C30	−176 (5)
C2—N3—C11—C10	0.4 (7)	C22—N23—C31—C30	1 (7)
C4—N3—C11—C12	0.6 (9)	C24—N23—C31—C32	−7 (7)
C2—N3—C11—C12	179.2 (7)	C22—N23—C31—C32	170 (5)
C9—C10—C11—N3	178.6 (5)	C29—C30—C31—N23	177 (7)
S1—C10—C11—N3	−1.5 (6)	S2—C30—C31—N23	−4 (8)
C9—C10—C11—C12	−0.1 (11)	C29—C30—C31—C32	8 (11)
S1—C10—C11—C12	179.8 (7)	S2—C30—C31—C32	−173 (4)
N3—C11—C12—C6	0.0 (12)	N23—C31—C32—C26	11 (7)
C10—C11—C12—C6	178.6 (6)	C30—C31—C32—C26	179 (5)
N3—C11—C12—C7	−179.5 (8)	N23—C31—C32—C27	−174 (4)
C10—C11—C12—C7	−0.8 (14)	C30—C31—C32—C27	−6 (9)
C5—C6—C12—C11	−0.5 (12)	C25—C26—C32—C31	−14 (6)
C5—C6—C12—C7	178.8 (10)	C25—C26—C32—C27	171 (5)
C8—C7—C12—C11	0.0 (16)	C28—C27—C32—C31	−5 (8)
C8—C7—C12—C6	−179.4 (10)	C28—C27—C32—C26	169 (5)

### Hydrogen-bond geometry (Å, º)

**Table:**

*D*—H···*A*	*D*—H	H···*A*	*D*···*A*	*D*—H···*A*
O1—H11···Cl1	0.84	2.34 (1)	3.174 (2)	174 (2)
O1—H12···Cl1^i^	0.84	2.40 (1)	3.240 (2)	178 (2)

Symmetry code: (i) −*x*+3/2, *y*−1/2, −*z*+3/2.
